# Physiological and Performance Impacts After Field Supramaximal High-Intensity Interval Training With Different Work-Recovery Duration

**DOI:** 10.3389/fphys.2020.01075

**Published:** 2020-10-08

**Authors:** Leandro Henrique Albuquerque Brandão, Thaysa Passos Nery Chagas, Alan Bruno Silva Vasconcelos, Vivian Conceição de Oliveira, Leonardo de Sousa Fortes, Marcos Bezerra de Almeida, Raquel Simões Mendes Netto, Fabrício Boscolo Del-Vecchio, Ezequias Pereira Neto, Leury Max Silva Chaves, David Jimenez-Pavón, Marzo Edir Da Silva-Grigoletto

**Affiliations:** ^1^Post-Graduate Program of Physical Education, Federal University of Sergipe, São Cristovão, Brazil; ^2^Functional Training Group, Federal University of Sergipe, São Cristovão, Brazil; ^3^Post-Graduate Program of Physiology Science, Federal University of Sergipe, São Cristovão, Brazil; ^4^Department of Physical Education, Federal University of Paraiba, João Pessoa, Brazil; ^5^School of Physical Education, Federal University of Pelotas, Pelotas, Brazil; ^6^MOVE-IT Research Group, Department of Physical Education, Faculty of Education Sciences, University of Cádiz, Cádiz, Spain; ^7^Biomedical Research and Innovation Institute of Cádiz (INiBICA) Research Unit, Puerta del Mar, University Hospital University of Cádiz, Cádiz, Spain

**Keywords:** metabolism, biochemistry, interval training, blood lactate, creatine kinase

## Abstract

High-intensity interval training (HIIT) has numerous external load control variables. The management of these variables makes the physiological responses and performance presented by athletes also modify. The present study aimed to assess the activity of CK and LDH enzymes, performance and metabolic responses caused by two HIIT protocols above the maximum in male recreational runners. Fifteen recreational male runners performed two HIIT protocols in randomized order with multiple conditions: 1) H15 (*n* = 15), with a HIIT protocol of 15:15 work-recovery duration, and 2) H30 (*n* = 15) with a HIIT protocol of 30:30 work-recovery duration. Both protocols were performed at similar intensity (130% vV̇O_2__*max*_), one set until voluntary exhaustion. Blood samples were collected and used to capture the levels and activities of blood lactate (BLac: mmol⋅L^–1^), glucose (GLU: mg⋅dL^–1^), creatine kinase (CK: U⋅L^–1^), and lactate dehydrogenase (LDH: U⋅L^–1^). BLac and GLU were collected at pre, five, and ten minutes after the H15 and H30 protocols were performed. Blood samples were used to measure the activities of CK and LDH enzymes, which were verified 24 h before and 48 h after the protocols. The distance traveled (m), total time (s), and bouts performed (rep) were also registered. Significant differences between conditions H15 and H30 were observed in the bouts performed (*p* = 0.001; ES = 1.19). Several statistical differences were found over time for BLac [pre vs. post 5 (both conditions: *p* = 0.001), pre vs. post 10 (both conditions: *p* = 0.001), and post 5 vs. post 10 (H30: *p* = 0.004)], CK [pre vs. post 24 (H15: *p* < 0.001; ES = 0.97 and H30: *p* = 0.001; ES = 0.74) post 24 vs. post 48 (H30: *p* = 0.03; ES = 0.56)], and LDH [pre vs. post24 (H15: *p* = 0.008; ES = 1.07 and H30: *p* = 0.022; ES = 0.85). No statistical differences between conditions were observed for any blood parameter. Thus, the volunteers exhibited equal performance in both protocols, which resulted in a similar physiological response. Despite this similarity, in comparison to H15, the H30 protocol presented lower CK activity post 48 and lactate levels after 10 min post protocol.

## Introduction

High-intensity interval training (HIIT) is a training strategy that intersperses short periods of high intensity exercise, most often cyclical (i.e., cycling and running), with stages of passive or active recovery (Billat, 2001; Gibala et al., 2006; Buchheit and Laursen, 2013a; Tschakert and Hofmann, 2013). HIIT is a unique protocol that is chosen by recreational endurance runners to improve their sports performance in a shorter period since it provides a significant intensification in the performance of athletes (Milanovic et al., 2015; Garciá-Pinillos et al., 2017).

In a HIIT protocol, the control of the training dose can be performed through variables related to the different sessions (i.e., set number, recovery between sets, total time training) and variables related to specific intervals (Buchheit and Laursen, 2013a). Regarding interval variables, control of work duration and recovery are largely based on the objectives of the training process and the protocols used, and the time or the work/recovery ratio adopted in the session can be modified (Buchheit and Laursen, 2013b; Tschakert and Hofmann, 2013; Gillen and Gibala, 2014). Stimuli that have the same work-recovery ratio (W:R ratio) and that differ in terms of the duration of the exercise and recovery intervals may show different neuromuscular, physiological and metabolic responses, more accentuated in protocols with longer duration (Wakefield and Glaister, 2009; Gosselin et al., 2012; Cipryan, 2017).

The prescription of HIIT protocols for recreational road runners is based on several variables in training dose control (Buchheit and Laursen, 2013a), which, when manipulated, can promote different acute metabolic, physiological, and neuromuscular reactions (Buchheit et al., 2012; Gosselin et al., 2012; Cipryan, 2017; Green et al., 2017). Metabolic activity from different HIIT protocols with different work durations, recovery, and ratio combinations were previously compared. It was found that work interval duration was longer than recovery (i.e., W:R ratio 2:1) and that there were higher lactate values compared to protocols with lower (i.e., W:R ratio 1:1) (Gosselin et al., 2012). However, and conclusions on the impact of HIIT methods using only the blood lactate concentration are uncertain, due to the production-release-metabolization dynamics of this variable (Laursen and Buchheit, 2019).

Gosselin et al. (2012) have reported that cardiovascular and metabolic responses are promoted by four submaximal HIIT protocols with different work and recovery ratios (90/30; 60/30; 30/30, and 60/60). The highest values of lactate concentration and rating of perceived exertion were observed in their protocols that had a longer duration of the determination. However, it has been highlighted that stimuli with similar ratio (60/60 and 30/30) did not provide significant physiological/metabolic differences, probably due to the use of submaximal intensities, which could be considered as a limitation in the prescriptions of HIIT with 1:1 W:R ratio (Buchheit and Laursen, 2013b) to individuals.

On the other hand, the acute responses of three HIIT protocols performed at maximum intensity (100%V̇O_2__*max*_), and the same W:R ratio (15/15; 30/30; 60/60) on blood markers to mediated biochemical markers abnormalities in moderately trained individuals have been observed (Cipryan, 2017). High concentrations of Lactate Dehydrogenase (LDH) and Creatine Kinase (CK) in blood plasma, which are considered as indicators of muscle damage (Brancaccio et al., 2008) caused by exercise, were observed immediately, three and 24 h after performing the protocols (Cipryan, 2017). In this context, when comparing sessions of 30/30 and 60/60 to 100% of vV̇O_2__*max*_, higher effort duration is associated with greater physiological stress(Farias-Junior et al., 2019).

In terms of work duration, the intensity at which HIIT is performed is an important response modulator for this training method (Wood et al., 2016). Wakefield et al. (Wakefield and Glaister, 2009) verified the metabolic responses and performance at intervals of 20, 25, and 30 seconds performed at 105% e 115% V̇O_2__*max*_, with 20 seconds of recovery and observed higher values of lactate in the longest protocols (30 s) when performed at greater intensity (115% V̇O_2__*max*_). The study found that when the protocols that had a shorter duration were performed, the individuals had a higher number of repetitions and time to exhaustion.

It is important to emphasize that several similar studies, including ours, compared different protocols on a treadmill. There are differences between running on a treadmill and in the field and it is important to consider the biomechanical nature of both situations. These divergences lead to changes in muscle fiber activation patterns and possibly promote different physiological responses, such as metabolic response and enzymes related to tissue damage (CK and LDH) (Miller et al., 2019). Few studies have investigated the CK and LDH activities in supra-maximum HIIT protocols under field conditions, which need to be better understood in relation to the impact these scenarios have on recreational runners.

To the best of our knowledge, few studies have attempted to verify which physiological impacts (CK and LDH activities and metabolic responses) and the performance presented by endurance road runners when performing one set of HIIT at the supramaximal intensity with a similar W:R ratio (1: 1) and different duration of work and recovery (30:30 and 15:15). Therefore, the present study aimed to assess the activity of CK and LDH enzymes, performance, and metabolic responses caused by two HIIT protocols above the maximum in recreational runners. The subsequent findings of this study could aid coaches and training experts, helping them to understand the workload that HIIT protocols can provide to recreational runners. Given the similarity of both protocols, we hypothesized that the performance of volunteers would be similar, however, due to the higher number of bouts of H15 protocol performed, it was anticipated that there would be an increased enzymatic activity of CK and LDH in participants.

## Materials and Methods

A crossover, counter-balanced, and randomized study with repeated measures was conducted. The anthropometric, blood, and performance evaluations of participants were made on an official outdoor athletics running track, through seven visits to the Federal University of Sergipe. The study was approved by the UFS Human Research Ethics Committee under the number 3.709.418.

### Participants

Sample size calculation was performed using the G^∗^Power version 3.1.9.2 software, assuming a difference of 37.79 U⋅L^–1^ for CK, based on a previously published study (Cipryan et al., 2017). A statistical power of 80% was used to confirm a sample size of *n* = 13 individuals for both conditions. The following criteria were considered: (a) we included participants with at least six months of experience road running; (b) all participants were non-smokers; (c) the included participants did not take anti-inflammatory drugs in the two weeks before the evaluation period; (d) and we excluded potential participants who experienced musculoskeletal disorders, heart, or lung diseases during the evaluation period. We also excluded individuals who presented any disorder or discomfort during the evaluation and protocols and who did not complete some evaluations, as well as those who ingested alcoholic beverages in the 48 h before the evaluation. Further to this, we also excluded any participants who consumed any or all dietary supplements composed of vitamins, minerals, or bioactive compounds, as well as caffeine, topical corticosteroids, or aspirin.

Fifteen healthy and recreational male runners were selected for the study (see characteristics in Table 1). All participants were instructed not to perform vigorous exercises 48 h before the first visit (performance evaluation) and throughout the study to avoid interference in performance and blood tests. The evaluations were carried out individually in the afternoon, between 3 and 5 pm, with each session lasting at least one hour for each participant. The individuals were instructed to repeat the same diet, clothes, and shoes on the days when the H15 and H30 protocols were performed. All participants read and were informed about the research procedures, and signed an informed consent form. The procedures of this study were in accordance with ethical standards in sports science and exercise (Harriss et al., 2019).

**TABLE 1:** Participant characteristics (mean ± standard deviation).

Parameter (*n* = 15)	Mean ± Standard Deviation
Age (year)	28.0 ± 8.0
Weight (kg)	73.9 ± 17.5
Height (m)	1.7 ± 0.1
BMI (kg⋅m^2^) ^–1^	24.9 ± 4.8
Running experience (Months)	28.4 ± 22.8
Training frequency (days⋅week^–1^)	4.0 ± 1.0
VO_2__*max*_ (mL⋅kg^–1^⋅min^–1^)	51.4 ± 5.7
vV̇O_2__*max*_ (km⋅h^–1^)	14.2 ± 1.9
130% vV̇O_2__*max*_ (km⋅h^–1^)	18.5 ± 2.4
HR_*r*__*est*_ (bpm)	62 ± 7

*BMI, Body Mass Index; VO_2__max_, Maximum oxygen consumption; vV̇O_2__max_, Maximal aerobic velocity; HR_rest_, Heart rate rest.*

### Procedures

**FIGURE 1:**
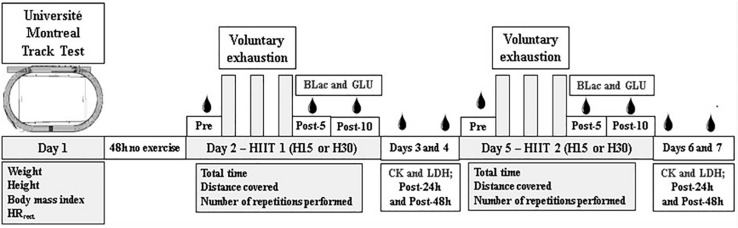
Description of the experimental study procedure. HR_*rest*_: Heart Rate Rest; BLac: Blood Lactate; GLU: Blood Glucose; CK: Creatine Kinase; LDH: Lactate dehydrogenase; H15: Work-recovery duration 15 s (15:15); H30: Work-recovery duration 30 s (30:30).

The initial assessment procedures were performed during the first visit, when anthropometric measurements (body mass, height, and body mass index), resting heart rate (HR_*rest*_) and vV̇O_2__*max*_ (indirectly determined) were verified. The HIIT protocols were performed during the second and fifth visit, in a randomly determined order, recording hemodynamic measurements (maximum [HR_*max*_], average heart rate [HR_*mean*_]), performance (total stimulus time, traveled distance, and sprints performed), metabolic parameters (lactate [BLac]), and glucose [GLU]). The HIIT protocols were performed on days two and five (see Figure 1), with an interval of 72 hours between them.

Plasma blood samples were used to determine metabolite concentrations. The samples were obtained before and then at periods of five and ten min after participants had undertaken the experimental HIIT protocols. Blood was also then collected two days after HIIT protocols, to determine how they affected CK and LDH activities.

*Anthropometric assessment:* Body mass and height were recorded on the first visit using an analog scale and a coupled stadiometer of the Welmy^TM^ (Santa Bárbara d’Oeste, São Paulo, Brasil) with a 100 g and 1.0 cm precision scale, respectively. Calculation of the body mass index (BMI) was also performed using the equation: [body mass (kg)⋅height^2^ (m)]^–1^.

*Heart Rate measurements:* Evaluation of vV̇O_2__*max*_ was initiated by checking the resting heart rate (HR_*rest*_). The HR_*rest*_ was registered in the sitting position after the individual wore a cardiofrequency meter with a chest strap for five minutes (Garmin Forerunner 910XT^TM^, Lenexa, Kansas, United States). The lowest beats per minute were noted as HR_*rest*_. During the evaluation of the HIIT protocols, the maximum and average heart rate was also verified using the same device and recorded at the end of the protocols, according to the values presented by each individual.

*vV̇O_2__*max*_ assessment:* To evaluate vV̇O_2__*max*_, the progressive test the “Université de Montréal Track Test” (UMTT) was used on an athletics track made with synthetic material (Leger and Boucher, 1980). This UMTT presents reliable measures for evaluating vV̇O_2__*max*_ and estimating V̇O_2__*max*_, as verified by another study (Leger and Boucher, 1980), which showed positive correlations values *r* = 0.97 and a difference of 0.03 ± 0.05 m between the two measurements and was used to study recreational runners (Jiménez-Pavón et al., 2015). The test was started at a speed of 7 km⋅h^–1^ with increments of 1 km⋅h^–1^ every two min of running, as previously described (Souza et al., 2014). The evaluation ended when the individual was unable to reach the sound signal three times in a row, with its passage through the respective cone.

The estimation of maximum oxygen consumption (V̇O_2__*max*_) was calculated from the equation below (Souza et al., 2014):

VO(mL⋅kg⋅-1min)-12max=0.0324x+22.134x+14.49

In the equation, the “x” value corresponds to the speed (km⋅h^–1^) reached in the last stage completed by each participant in the incremental test, which corresponds to the individual’s vV̇O_2__*max*_ (Leger and Boucher, 1980). The HIIT protocols intensity was determined through the equation below.

Intensity=(vVOx2max0.30)+vVOmax2

### Description of HIIT Protocols

The experimental protocols were performed on two separate days, which allowed for a minimum recovery interval of 72 h up to 7 days (see description of protocols in Table 2) at a working and recovery ratio of 15:15 (H15) and 30:30 (H30). The session started with a five-minute warm-up as part of which individuals ran 800 meters at a speed of 9.6 km⋅h^–1^ controlled through the average time the individual performed the activity. At the end of the warm-up and before the beginning of the HIIT protocol, the athletes were equipped with a heart rate monitor and we explained how the protocol would be carried out in practice, which lasted an average of three min. The subjects were encouraged to perform as many stimuli as possible in both protocols. The detailed description of the protocols can be seen in Table 2. HR was monitored during the execution of the HIIT protocols.

**TABLE 2:** Description of the control variables, maximum and average heart rate presented by the individuals in the HIIT protocols H15 (15:15) and H30 (30:30).

	H15	H30
Work intensity (% vV̇O_2__max_)	130	130
Work duration (s)	15	30
Recovery intensity	Passive	Passive
Recovery duration (s)	15	30
Work:recovery ratio (ratio)	1:1	1:1
Number of repetitions	Voluntary exhaustion	Voluntary exhaustion
Number of sets	1	1

The protocol intensity was controlled by calculating the maximum distance that the individual should run for 15 or 30 seconds with a speed of 130% of vV̇O_2__*max*_, (m⋅s^–1^) using the equation below. The participant was instructed to run an individual distance (D) for a corresponding time for the prescribed protocols (15 seconds for the H15 protocol and 30 seconds for the H30 protocol). Verbal encouragement for athletes to run at the correct speed was provided throughout the protocol.

D=130%vVOx2maxWorkTime(15or30seconds)

The execution of the protocols was interrupted when the individual was unable to complete the distance in the stipulated time for three consecutive times or voluntarily decided not to continue with the execution of the protocol.

### Blood Analysis

We collected blood from brachial veins in tubes with EDTA (4 mL) through a vacuum capture system, before the beginning of the HIIT protocols, and five and 10 min after the end of the exercises. Moreover, blood samples with EDTA (4 mL) and Serum (4 mL) were also collected 24 and 48 h after each HIIT session. Plasma and serum were obtained through centrifugation of blood collected at 1500 rpm at 4°C for 10 min and used to determine the enzyme’s creatine kinase (CK) and LDH, respectively.

The verification of the plasma concentrations for lactate and glucose were performed on blood plasma. The blood glucose enzymatic determination procedures were performed using a semi-automatic and spectrophotometric analyzer Bioplus 2000^TM^ (Bioplus Produtos para Laboratórios Ltda, Barueri, São Paulo, Brasil). The analysis of CK, LDH, and lactate concentrations were performed using the plate spectrophotometer (BIOTEK Instrument Model Synergy 2^TM^, Winooski, VT, United States). All metabolite and enzyme analysis procedures were performed following the instructions provided by the reagent manufacturer.

### Statistical Analysis

Data were presented as means and standard deviations. The verification of normality and homogeneity were performed using the Shapiro-Wilk and Levene tests, respectively. Comparisons of the biochemical parameters between moments and protocols were carried out through analysis of variance of repeated measures (ANOVA 2 × 3), which were complemented by Bonferroni’s *post hoc*. The T-student test for paired samples was also used to analyze the differences between conditions in performance variables.

Simple linear regression analyses were used to analyze the association between HIIT performance (sprints, total stimulus time, and distance as independent variables) and changes (baseline to post 24) in biochemical parameters by HIIT protocol. As the independent variables were only associated with the pure regression and change in CK, a multiple linear regression model was built to analyze the associations of these independent variables with the change in CK. After an evaluation of collinearity among independent variables, only distance and total time were kept in the model, where b = regression or determination coefficient and B = standardized coefficient.

The effect size (ES) was calculated according to the proposed guidelines (Rhea, 2004), assuming the classification table for recreational individuals as parameters to determine the level of the effect (<0.35 for trivial effect size; 0.35 - 0.80 for small effect size; 0.80 - 1.50 for moderate effect size; > 1.5 for high effect size). We also calculated the absolute difference (Δ) and relative (Δ%) between the values. All statistical procedures were performed using SPSS software version 23. A significant level was set at *p* < 0.05.

Magnitude-based inference statistics were calculated using spreadsheets available on the website: http://sportsci.org/2006/wghcontrial.htm27. The probability that some of the protocols will change positively (increase), null or negative (decrease) was calculated and assessed qualitatively as follows: < 1% trivial (almost certainly not); 1–5% very unlikely; 5–25% unlikely; 25–75% possible; 75–95% likely; 95–99% very likely and > 99% almost certainly yes. We considered values < 5% as inconclusive (Batterham and Hopkins, 2006; Abad et al., 2016).

## Results

Fifteen male recreational trained athletes completed the assessment of vV̇O_2__*max*_ and, subsequently, the two protocols proposed for the study. No participant reported malaise or was excluded from the procedures due to absence, and all included participants completed all protocols and attended each stage of evaluation.

### Performance

**FIGURE 2:**
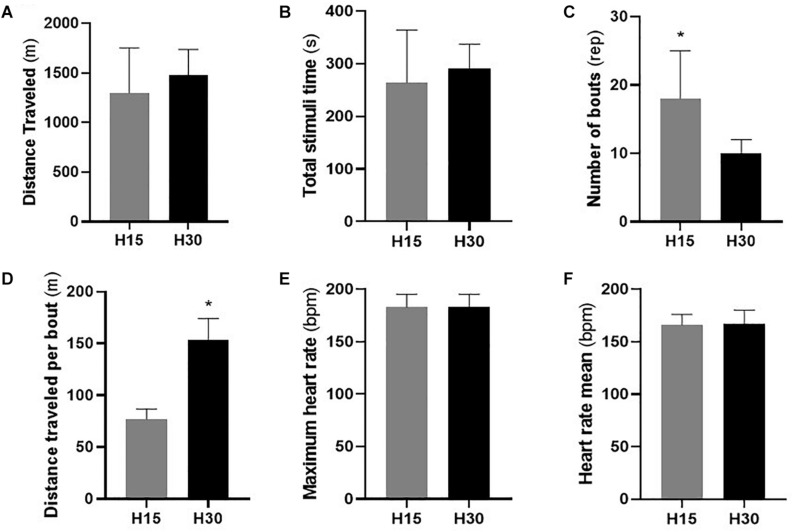
Performance values evaluated by the traveled distance **(A)**, total stimulus time **(B)**, the number of repetitions performed **(C)**, distance traveled per bout **(D)**, maximum **(E)**, and average **(F)** heart rate presented in the protocols H15 (W:R ratio 15:15) and H30 (W:R ratio 30:30). bpm: beat per minute. Values are mean ± standard deviation. (*) Statistically significant difference (*p* < 0.05).

Performance values, average distance, HR_max_ and HR_mean_ for the protocols are shown in Figure 2. Each runner’s performance was compared and they did not show significant differences in distance covered (*p* = 0.199; Δ% = 13.52; Δ = 175.77; ES = 0.67) or total time (*p* = 0.315; Δ% = 9.85; Δ = 26.00; ES = 0.55). However, when number of bouts were compared, the H15 protocol showed higher values (16.0 ± 6.0 bouts) compared to H30 (10.0 ± 2.0 bouts), showing a statistically significant difference (*p* = 0.002; Δ% = 45.08; Δ = 8.00; ES = 1.19).

### Blood Variables

Blood lactate increased immediately after participants had completed the HIIT protocols, and slightly decreased 10 min after they ended (see Figure 2). Over time, both HIIT protocols caused significant changes, as established by comparing pre vs. post5 (H15: *p* = 0.001 and ES = 23.07; H30: *p* = 0.001; ES = 17.34) and pre vs. post 10 (H15: *p* = 0.001 and ES = 21.14; H30: *p* = 0.001 and ES = 13.18). After 10 min, the H30 protocol showed significant reduction in blood lactate levels when compared to post 5 (*p* = 0.004; ES = 1.71). There was no statistically significant differences at any time for both protocols.

**FIGURE 3:**
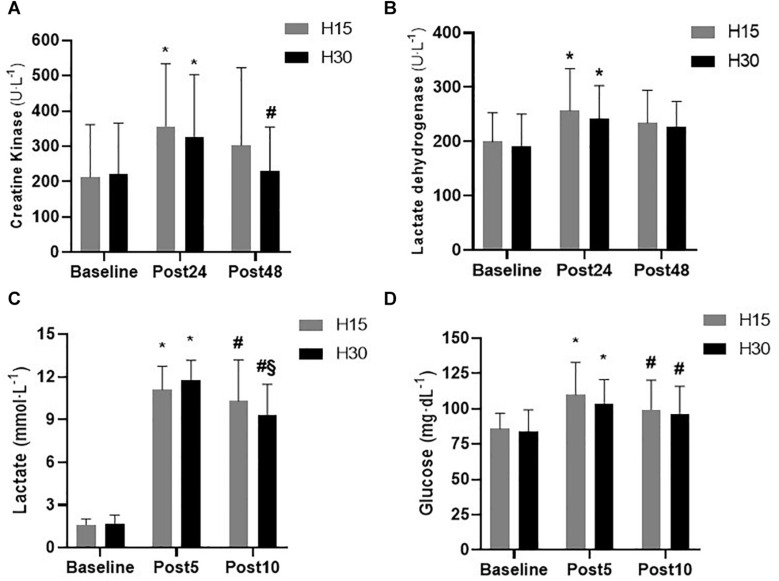
Muscle damage and metabolic responses assessed using CK **(A)**, LDH **(B)**, blood lactate **(C)**, and glucose **(D)** concentrations observed in volunteers evaluated in the protocols with work and recovery ratio of H15 (W:R ratio 15:15) and H30 (W:R ratio 30:30). Values are mean ± standard deviation. (*) Significant differences (*p* < 0.05) in the comparison between pre vs. post 24 and pre vs. post5. (#) Significant differences (*p* < 0.05) in the comparison between the post24 vs. post48 moments and post5 vs. post10. (§) Significant differences (*p* < 0.05) in the comparison between pre vs. post10.

Similar results were observed in the analysis of blood glucose concentration, with an increase in plasma concentrations five min after the protocols were performed, and subsequent reductions after ten min. Significant changes over time were found in both protocols after comparing pre vs. post 5 (H15: *p* = 0.002 and ES = 2.21; H30: *p* = 0.012; and ES = 1.28) and post 5 vs. post 10 (H15: *p* = 0.001 and ES = 0.70; H30: *p* = 0.022; and ES = 0.40). There was no statistically significant difference in conditions at any time (pre: *p* = 0.771; post 5: *p* = 0.427; and post 10: *p* = 0.735). Figure 3 illustrates the values of the lactate and glucose variables after the subjects performed the protocols.

We observed significant changes in CK, 24 h after the two HIIT protocols (H15: *p* < 0.001 and ES = 0.97; H30: *p* = 0.001 and ES = 0.74), and showed a reduction of concentrations in the post 48 sample for the H30 condition only (*p* = 0.03; ES = 0.56; Δ% = −29.87) (see Figure 3). There were no significant differences between conditions at any time.

Magnitude-based inference in the comparison between pre and post 24h samples demonstrated an increase that was “most likely positive” for CK after the H15 protocol. A “probably positive” increase was observed in the inference made for the H30 protocol on the same variable, as shown in Table 3.

**TABLE 3:** Mean and standard deviation (SD), relative change (Δ%) effect size (effect size) and inference based on magnitude of indirect blood markers.

Measures	Protocols	Δ%	Effect Size	Pre-post24h p value	% chance	Qualitative inference
CK (U⋅L^–1^)	H15	67.84	0.97	0.0001	98.9/1.1/00	Very likely positive
	H30	48.30	0.74	0.001	90/10/00	Likely positive
LDH (U⋅L^–1^)	H15	28.75	1.07	0.008	93.9/6.1/00	Likely positive
	H30	26.40	0.85	0.022	84.7/15.3/0.1	Likely positive

*CK, Creatine Kinase; LDH, Lactate dehydrogenase.*

A significant increase in LDH concentrations over time was observed 24 h after protocols for both conditions (H15: *p* = 0.008 and ES = 1.07; H30: *p* = 0.022 and ES = 0.85). Significant differences were not seen in the other comparisons over time and between conditions at any time (see Figure 3).

The qualitative inference was also calculated for the LDH responses presented by the individuals. Regarding the pre vs. post 24 comparisons in both conditions, the qualitative assessment was “probably positive” for LDH modification after the protocols were performed, according to Table 3.

**FIGURE 4:**
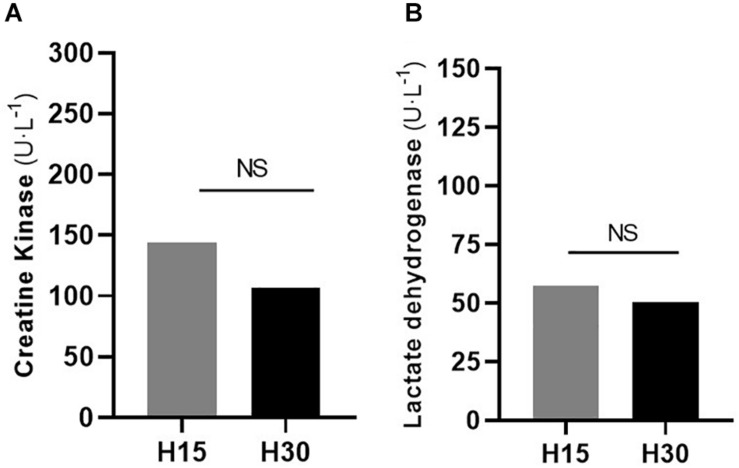
Absolute difference (post 24 – pre) assessments in the H15 (W:R ratio 15:15) and H30 (W:R ratio 30:30) protocols for the evaluation of muscle damage verified through the concentrations of CK **(A)** and LDH **(B)**. CK: Creatine kinase; LDH: Lactate dehydrogenase. Mean values. (*) Significant differences (*p* < 0.05). (NS): Not statistically different in comparison between protocols.

Values of absolute difference (post 24-pre) of the CK and LDH markers are shown in Figure 4. Despite the differences observed in the H15 protocol, statistically significant differences between conditions (H15 vs. H30) in CK measures (*p* = 0.205; ES = 0.34; DIF% = 35,22%) were observed. A similar observation was made by the LDH in the same comparison (*p* = 0.789; ES = 0.09; DIF% = 6.87).

**FIGURE 5:**
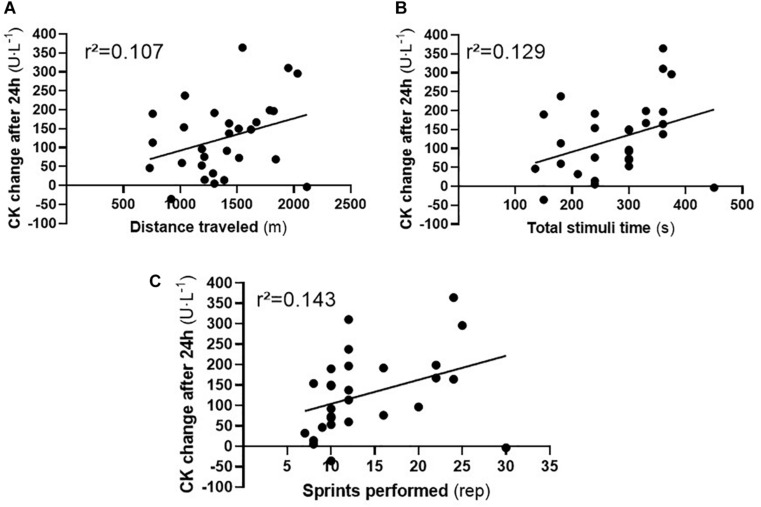
Individual correlation value between change in CK concentrations after 24h and performance variables traveled distance **(A)**, total time **(B)**, and performed sprints **(C)**. CK: Creatine kinase. Note: CK: Creatine kinase.

An evaluation of the correlation indicated a positive, low (*r* = 0.378), and significant (*p* = 0.020) correlation between CK change and the number of sprints performed (*r*^2^ = 0.143). A similar result was observed when the other independent variables were verified in relation to the change in CK (total time: *r* = 0.359; *p* = 0.026; *r*^2^ = 0.129 and distance traveled: *r* = 0.327; *p* = 0.039; *r*^2^ = 0.107), as seen in the Figure 5. Linear regression analyses showed an independent and significant association between total time and distance traveled with change in CK (*b* = 0.194; *B* = 0.609; *p* = 0.016 and *b* = 1.274; *B* = 0.731; *p* = 0.002, respectively) for H30 but not for H15 protocol. The multiple regression model explained 55% (*p* = 0.008) of the variance in the change of CK in the H30 protocol.

## Discussion

The present study aimed to assess the activity of the CK and LDH enzymes, performance, and metabolic responses caused by two HIIT protocols above the maximum in male road runners. The findings of this study showed similar performance values for individuals in the H15 and H30 protocols. Moreover, the difference between the relative changes (Δ%) were greater for H15 compared to H30 in the activity of the CK enzyme, an important finding since H15 performed similarly when compared to H30.

### Performance

As previously described, both protocols were performed on an outdoor athletics track and this may have influenced the performance of individuals (Miller et al., 2019). HIIT protocols performed in external environments possess periods of acceleration and deceleration to start and end in each performed stimulus. These characteristics initiate important muscle actions that increase energy expenditure during running activity (Bisciotti and Sagnol, 2000).

The recovery time is an important factor for the similar performance of the protocols since supra-maximum intensities require longer recovery time between work intervals to achieve high performances (Germano et al., 2019). Shorter recovery time after supramaximal intensity sprints seem to have an important impact on neuromuscular fatigue, which impair performance due to the short duration of recovery, not enough to remain at a high intensity (Schoenmakers et al., 2019).

Moreover, shorter recovery time also influenced the ability to restore creatine phosphate (PCr) stocks and myoglobin and skeletal muscle reoxygenation, which would have affected the performance of participants in the H15 protocol (Zafeiridis et al., 2010). Thus, recovery times < 30 seconds seem to impair the organic potential for energy production in subsequent supra-maximal sprints (Schoenmakers et al., 2019).

### Blood Variables

After performing H15 and H30, individuals possessed higher concentrations of lactate and glucose in the post-5 period compared to pre-HIIT, which did not show a statistically significant difference when compared to each other. This fact demonstrated the high activity of the anaerobic system for ATP resynthesize in both protocols since the intensity reactions were similar for both HIIT (Gosselin et al., 2012; Cabral-Santos et al., 2015).

The recovery time of the protocols (which differed between them) may have influenced the reduction in the lactate concentration observed 10 min after the end of the application of the H30 protocol (Germano et al., 2019). At the time post-10, BLac levels began to decrease in both groups, which is shown in the literature to be the period when there is a drop in the concentrations of this metabolite (Saltin et al., 1976; Cipryan et al., 2017). However, there was a significant decrease observed after the H30 protocol. The longer duration of rest at H30 might have favored the rapid removal/use of lactate since 30 seconds seemed to be a time of rest that favored the timing/reuse of this metabolite by body structures (Saltin et al., 1976; Schoenmakers et al., 2019).

A different response was observed in relation to glucose, which returned close to baseline values 10 min after performing the protocols. Such a phenomenon could have occurred due to the process of transporting glucose molecules into muscle tissue by GLUT4 (glucose transmembrane transporter 4) protein. GLUT4 facilitated the use of glucose by the body muscles and enhance the energy homeostasis and actions (Abderrahman et al., 2016).

Regarding CK, the H15 protocol showed the greatest absolute difference (Post24 - Pre), effect size, and magnitude-based inference rating compared to H30 concerning the CK and LDH variables. However, linear regression showed an independent and significant association between total time and the distance traveled with H30. Performance variables explain 55% of the change in CK 24h after the H30 protocol, which can be considered an important explanation for the main characteristics of this study. Gastin et al. (2019) demonstrated results in related performance factors for Australian football and witnessed changes in plasma CK levels after a match. The authors observed that the distance covered contributed to the significant changes in player CK after the Australian football match (about 15%). Moreover, the authors found that accelerations, decelerations, and impacts during races contributed to a more significant change of CK in sports activities since it requires high physical contact (Gastin et al., 2019). It is important to highlight that increased CK and LDH can show a strength related to impaired neuromuscular performance (Farias-Junior et al., 2019; Gastin et al., 2019). The increased CK and LDH 24 h after the HIIT session may indicate the need for a change in external training load in the following training session.

The present study showed a significant increase of 24 h duration after the protocols and a low and significant correlation was observed between CK change and the performance variables (the number of sprints performed being one of them). A report by Thorpe et al. (Thorpe and Sunderland, 2012) showed a strong linear correlation (*r* = 0.80; *r*^2^ = 0.646) and statistical significance (*p* = 0.029) between the number of sprints performed and CK change (Thorpe and Sunderland, 2012). The study also evaluated the difference and factors that contributed to a change in CK concentrations after a soccer match in amateur players and found no statistically significant differences when comparing the pre-measures with measures after the match. The wide variation in intensity that occurs in sprints during soccer matches might be the reason for the high correlation found by Thorpe et al. (Thorpe and Sunderland, 2012). Inter-individual variability, intensity, session volume, type of exercise evaluated, and the level of sports activity can influence the CK change in a given exercise (Baird et al., 2012; Cipryan, 2018).

A large number of accelerations and decelerations have been linked to an increase in biochemical markers CK, LDH, and inactivity (Thorpe and Sunderland, 2012). Eccentric muscle activity is associated with greater muscle damage (Proske and Morgan, 2001), as caused by acceleration and deceleration actions in high-speed running activities (Thorpe and Sunderland, 2012; Gastin et al., 2019), which are characteristically situated in the present study. The eccentric muscle contraction, observed in both protocols contributed to an increase in tension on the muscle, inducing the rupture of structural components of the muscle, which contributed to the increase in plasma CK after 24 h. Thus, a greater number of sprint repetitions were performed in the H15 protocol, which provided a greater number of accelerations and decelerations compared to H30, causing a greater demand for the activation of muscles that perform eccentric contractions during a run, triggering a response in the markers of CK (Armstrong et al., 1983; Thorpe and Sunderland, 2012; Gastin et al., 2019).

A significant reduction in CK concentration was observed 48 h after the H30 protocol. A similar response was presented in a study that assessed the influence of deceleration on muscle damage variables after 24 and 48 h of the application of the protocols (Minahan et al., 2018). In this study, Minahan et al. evaluated 14 moderate trained individuals, who participated in team sports and underwent two treadmill tests that required the action of deceleration, in order to verify the influence of this component on vital metabolic markers and muscle damage. The authors observed a significant reduction in the concentrations of LDH after 48 h and a decreased level of LDH was found when the protocol was used with the deceleration condition, demonstrating that protocols that do not have these components tend to reestablish the concentrations of these markers, meaning a person can resume sports activities with minimal LDH concentration after 48 h. In the present study, LDH did not show any reduction 48 h after the application of the protocols, despite showing a significant increase after 24 h. This phenomenon may be associated with the intensity of the modification of LDH activity, which was similar between the applied conditions in the present study (Cipryan, 2017).

To the best of our knowledge, this is the first study comparing metabolic responses, the activities of CK and LDH enzymes, and performances of two HIIT protocols (performed during voluntary exhaustion on the running tracks). The current findings show that although participants performed more sprints in the H15 protocol, the individuals showed faster recovery after H30, with a significant reduction after 48h. However, a similar performance was observed when the distance traveled and the total time used were compared, enabling new thinking and applicability of HIIT protocols on the running tracks.

There are some limitations to these results. Neuromuscular performance measures were not measured and could be used to ensure recovery between protocols and to verify the neuromuscular impact of a HIIT session on individuals. In many ways, these measures are unnecessary, since the minimum 72 hour rest seemed to be sufficient to reestablish neuromuscular recovery after the application of this mode of exercise (Minahan et al., 2018; Fiorenza et al., 2019) and it is known that HIIT promotes central and peripheral fatigue, meaning that such an observation is not crucial for the present study (Marqués-Jiménez et al., 2017). Another limitation was the lack of Visual Analog Scale (VAS) to assess delayed onset muscle soreness (DOMS) after (e.g., time-course effect) experimental conditions as an additional indicator the assessment of muscle damage since CK can vary widely and is associated with certain situations that are not associated with exercise stress (i.e., inflammation from strokes unrelated to exercise). Despite being widely used as a marker of muscle damage, the activity of the CK enzyme is not only linked to physical exercise itself, which is an important limitation on its use in sports science (Brancaccio et al., 2008).

As a practical application, this study presents important insights about the workload caused by a set of different supramaximal HIIT protocols (carried out until voluntary exhaustion). Thus, highlighting that when HIIT with a 1:1 W: R ratio and shorter protocols (i.e., 15 seconds) might require a longer recovery period between sets. This may enable the runner to recover baseline levels of vital biomarkers associated with the internal load. Under these conditions, the performances of recreational runners were not similar when two HIIT protocols with the same W: R ratio were performed. However, future studies evaluating this perspective could further clarify these differences and establish the W: R ratio as an important modulating component of the HIIT workload.

## Conclusion

In conclusion, the present study demonstrated the modifications in the duration of the work intervals and recovery in HIIT protocols with equal intensity performed on the track until exhaustion can provide similar performance in recreational road runners. Metabolic responses to the H15 and H30 protocols also showed similar values after 5 and 10 min however H30 showed a reduction in lactate concentrations after 10 min, evidencing the important role of the duration of recovery during the protocols.

In addition, it is also concluded that regardless of the duration of the stimulus performed, in protocols performed on the track and until exhaustion, individuals increased the concentrations of vital markers associated with the activities of CK and LDH enzymes. Greater absolute difference between pre and post 24 presented by H15 for CK, as well as a significant decrease in CK concentrations in 48 h after H30, may demonstrate the role of deceleration as main factor for increasing the concentration of this enzyme in running athletes, in the present study.

## Data Availability Statement

The raw data supporting the conclusions of this article will be made available by the authors, without undue reservation.

## Ethics Statement

The studies involving human participants were reviewed and approved by CHuman Research Ethics Committee at the Federal University of Sergipe. The patients/participants provided their written informed consent to participate in this study.

## Author Contributions

LB wrote the article in its entirety. TC has effectively contributed to the correction and modification of all topics in the article. AV contributed in guiding and improving the exposition of ideas in the abstract, introduction and discussion topics. In addition, the author also contributed to the analysis of biochemical variables. VO contributed in guiding and improving the exposition of ideas in the introduction and methods topics. In addition, the author also contributed to research data collection. FD-V contributed in guiding and improving the exposition of ideas in all the article sections. In addition, the author also contributed to the correction of the entire manuscript. DJ-P and LF contributed in guiding and improving the exposition of ideas in all the article sections. In addition, the authors LC, MA, RM, and EN contributed to the correction of the entire manuscript after reviewers’ considerations. MDS-G was responsible for the final revision of the entire manuscript from the abstract to the references, as well as the authorization for its submission in this esteemed journal. All authors contributed to the article and approved the submitted version.

## Conflict of Interest

The authors declare that the research was conducted in the absence of any commercial or financial relationships that could be construed as a potential conflict of interest.
